# Instrumented gait analysis defines the walking signature of *CACNA1A* disorders

**DOI:** 10.1007/s00415-021-10878-y

**Published:** 2021-11-09

**Authors:** Elisabetta Indelicato, Cecilia Raccagni, Sarah Runer, Julius Hannink, Wolfgang Nachbauer, Andreas Eigentler, Matthias Amprosi, Gregor Wenning, Sylvia Boesch

**Affiliations:** 1 Center for Rare Movement Disorders, Department of Neurology, Medical University of Innsbruck, Anichstrasse 35, 6020 Innsbruck, Austria; 2 Neurobiology Division, Department of Neurology, Medical University of Innsbruck, Anichstrasse 35, 6020 Innsbruck, Austria; 3 Department of Neurology, Regional General Hospital, Lorenz Boehler Strasse 5, 39100 Bolzano, Italy; 4 Portablies HealthCare Technologies GmbH, Henkestr. 91, 91052 Erlangen, Germany

**Keywords:** *CACNA1A*, Gait analysis, Wearable sensors, Familial hemiplegic migraine type 1, Episodic ataxia type 2

## Abstract

**Background:**

Gait disturbances are a frequent symptom in *CACNA1A* disorders. Even though, data about their severity and progression are lacking and no *CACNA1A*-specific scale or assessment for gait is available.

**Methods:**

We applied a gait assessment protocol in 20 ambulatory patients with genetically confirmed *CACNA1A* disorders and 39 matched healthy controls. An instrumented gait analysis (IGA) was performed by means of wearable sensors in basal condition and after a treadmill/cycloergometer challenge in selected cases.

**Results:**

*CACNA1A* patients displayed lower gait speed, shorter steps with increased step length variability, a reduced landing acceleration as well as a reduced range of ankle motion compared to controls. Furthermore, gait-width in patients with episodic *CACNA1A* disorders was *narrower* as compared to controls. In one patient experiencing mild episodic symptoms after the treadmill challenge, the IGA was able to detect a deterioration over all gait parameters.

**Conclusions:**

In *CACNA1A* patients, the IGA with wearable sensors unravels specific gait signatures which are not detectable at naked eye. These features (narrow-based gait, lower landing acceleration) distinguish these patients from other ataxic disorders and may be target of focused rehabilitative interventions. IGA can potentially be applied to monitor the neurological fluctuations associated with *CACNA1A* disorders.

## Introduction

*CACNA1A* disease spectrum encompasses a number of autosomal-dominant allelic disorders, which feature a various combination of episodic and chronic neurological signs [1]. Episodic manifestations include migraine with hemiplegic aura, attacks of paroxysmal ataxia, paroxysmal dystonia and epilepsy [1]. Chronic neurological symptoms consist of various degree of cerebellar ataxia, developmental delay, cognitive and psychiatric symptoms [1]. Based on the mutation type, prevalent phenotype and clinical course, four clinical entities are currently defined: familial hemiplegic migraine type 1 (FHM1), episodic ataxia type 2 (EA2) spinocerebellar ataxia type 6 (SCA6) and the early infantile epileptic encephalopathy type 42 (EIEE42) [1, 2].

Chronic cerebellar signs are a constant finding in SCA6 and develop in up to 70% of patients with FHM1 and EA2 [3, 4]. On the other hand, episodic manifestation are pathognomonic of FHM1 and EA2, but several SCA6 patients may also display paroxysmal vertigo at disease onset [5]. The most common signs at the examination are stance and gait ataxia, oculomotor abnormalities and dysarthria [3, 4]. This pattern reflects the prevalent involvement of cerebellar vermis as shown by imaging studies [6]. Especially in FHM1 and EA2, gait ataxia is usually mild. In our experience, several patients show a narrow-based gait but have for instance clear difficulties while performing the tandem gait or upon interferences. Natural history data on ataxia progression are available for SCA6 [7], but still lack in other *CACNA1A* disorders. In our experience, the rate of ataxia progression in FHM1 and EA2 is variable, but mostly milder than in other neurodegenerative cerebellar disorders. Furthermore, systematic assessment of gait disturbances in paroxysmal *CACNA1A* disorders are lacking.

Available clinical scales provide a semi-quantitative evaluation of gait and may not be able to capture subtle clinical benefit provided by physiotherapy or by future potential pharmacological therapies. For this reason, a prominent research effort of the past decades pointed to the development and validation of motion analysis technology. Importantly, instrumented gait analysis (IGA) by means of inertial measurement units has been demonstrated to be a feasible supplementary device with high sensitivity and specificity for assessing gait performance [8]. Detection systems consist of accelerometers and gyroscopes attached to shoes that record motion signals during standardized gait and leg function. They can be applied both as rapid assessment tools during the clinical examination in hospital and for home-based monitoring. Gait parameters defined by means of IGA can offer reliable, objective biomarkers that reflect subclinical changes in disease status, which may escape the naked eye.

Based on these considerations, we aimed at better characterizing the pattern of gait disturbances in *CACNA1A* disorders and at testing the IGA as objective assessment in this setting.

## Methods

### Study population

Genetically confirmed *CACNA1A* patients have been recruited at the Center for rare movement disorders of the Department of Neurology of Innsbruck. Patients could be recruited if (1) ambulant without aiding devices, (2) under a stable interval therapy (namely same posology of acetazolamide/flunarizine/4-aminopyridine in the 4 weeks preceding the examination). Exclusion criteria comprehended: (1) history or evidence of gait disorders not related to the underlying genetic disease (for instance status post stroke, major orthopedic diseases and further on), (2) current or past psychiatric disorders under neuroleptic therapy.

We recruited age- and sex-matched healthy controls among the personnel and the students of the Medical University of Innsbruck.

### Neurological examination and gait analysis

Collected clinical history included occurrence of falls in the 4 weeks preceding examination and fall-related injuries (since onset of disease). Physicians trained in evaluation of patients with ataxia (EI, WN, AE, MA or SB) performed the neurological examination and collected the standardized Scale for Assessment and Rating of Ataxia (SARA). The SARA consists of eight items evaluating ataxia in different tasks: gait, stance, sitting, speech, finger-chase, nose-finger test, fast alternating hand movements and heel-shin slide. The item “gait” assesses ataxia during regular walking and tandem gait. Its score ranges from 0 (no ataxia of gait) to 8 (unable to walk) points, with 4 points marking the transition to intermittent use of walking aid.

**Fig. 1:**
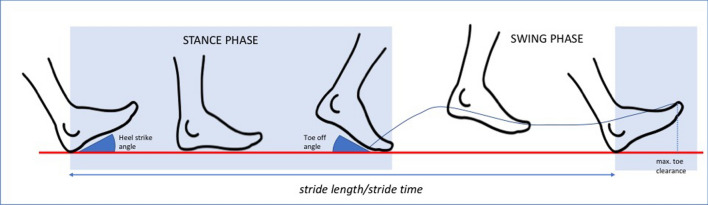
Gait parameters collected by means of instrumented gait analysis with wearable sensors

After the general examination, IGA was performed by means of wearable sensors (Shimmer Research Ltd., Dublin, Ireland), attached to the frontal part of both shoes. Spatiotemporal gait parameters (see Table 1 and Fig. 1) were calculated using pattern recognition algorithms [9], which have been previously validated from a technical and clinical point of view in patients with parkinsonian syndromes [10].

**Table 1:** Gait parameters collected by means of instrumented gait analysis with wearable sensors

Gait domain	Gait parameter	Definition
Pace	Gait speed (m/s)	Walking speed in the designated direction
	Stride time (s)	Duration of a gait cycle
	Stride length (cm)	Distance between two sequential point of stance contact of the same foot
Rhythm	Cadence (stride/m)	Stride rate per minute
	Swing phase (%)	Part of the gait cycle during which the foot has no contact with the ground
	Stand phase (%)	Part of the gait cycle during which the foot has contact with the ground
Fluidity	Heel-Strike angle (°)	Angle between foot and ground in the sagittal plane at heel-strike
	Toe-off angle (°)	Angle between foot and ground in the sagittal plane at toe-off
	Maximal toe clearance (cm)	Maximal toe height during the gait cycle
Maximal side excursion (cm)		Maximal side excursion from the line connecting two sequential points of stance contact of the same foot
Landing Impact Intensity (g)		Vertical acceleration at foot landing
Variability	Stride time CV (%)	Coefficient of variation of stride time
	Stride length CV (%)	Coefficient of variation of stride length
	Swing time CV (%)	Coefficient of variation of swing time

Patients and controls performed the gait tests wearing the IGA sensors in the same therapy hall of our Department, walking along a marked 10-m line back and forth during the first part of the afternoon. They were instructed to walk at self-determined preferred, fast, and slow speed, as well as while performing a concomitant “cognitive” task (sequential subtractions of 7 starting from 100). All gait tests were performed in the same sequence, without interruptions. If situations arose, that necessitated the repetition of a task, this was carried out once again. All patients completed the tests.

*CACNA1A* patients with current history of episodic symptoms underwent a second instrumented gait analysis after 10 min of walking on treadmill or cycling on an ergometer. Exclusion criteria for this additional task consisted of clinically significant heart or lung disease, history of severe attacks with disturbance of consciousness, history of falls-related injury.

### Data analysis and statistics

Gait parameters were extracted by means of dedicated machine learning algorithms [9]. Statistical analysis was performed by means of SPSS version 25. Data are reported either as mean ± standard deviation or median (interquartile range) according to their distribution, which was verified by means of Shapiro–Wilk test. Comparisons between patients and controls were performed by means of t-student or Mann–Whitney *U* test depending on data distribution. We applied Pearson´s and Spearman´s coefficient as correlation measures.

### Ethical issues

The present study was performed in accordance with the principles of the Declaration of Helsinki on research on human subjects and with the principles of good clinical and scientific practices. The ethic committee of the Medical University of Innsbruck approved the study (Study number 1128/2018). Each study participant gave written informed consent before inclusion in the study.

## Results

We recruited 20 genetically confirmed *CACNA1A* patients and 39 age- and sex-matched healthy controls. Demographic and clinical data of the *CACNA1A* cohort are summarized in Table 2. The study cohort comprehended 11 EA2, 5 FHM1 as well as 4 SCA6. Of the SCA6 subgroup, one patient was presymptomatic and another one just developed mild cerebellar signs (SARA score = 2). At the time of the examination, the median score in the gait subitem of the SARA was 2 (1;3), corresponding to a mild gait ataxia, with slight abnormalities in the standard gait and clear difficulties in tandem gait. Two patients had slight to moderate distal polyneuropathy. None of the patients had leg spasticity. Eleven patients displayed a nystagmus.

**Table 2:** Basic demographic and clinical data of the recruited *CACNA1A* cohort

Variable	*CACNA1A* patients (*n* = 20)
Age (years old)	52 ± 16
Sex (*n*, % female)	6 (30%)
Age at onset (years)	15 (5;53)
Disease duration (years)	17 (7;44)
Clinical course (*n*, %)	
Pure episodic Episodic with chronic cerebellar signs Chronic progressive cerebellar syndrome	4 (20%) 11 (55%) 5 (25%)
Attacks at present (*n*, %)	12 (60%)
Prophylaxis intake (*n*, %)	15 (75%)
MRI findings (*19 cases*) (*n*, %)	
No atrophy Isolated vermis atrophy Pancerebellar atrophy	1 (5%) 3 (16%) 15 (79%)

### Spatiotemporal gait parameters at baseline

Descriptive gait parameters and statistical comparisons are shown in Table 3.

**Table 3:**
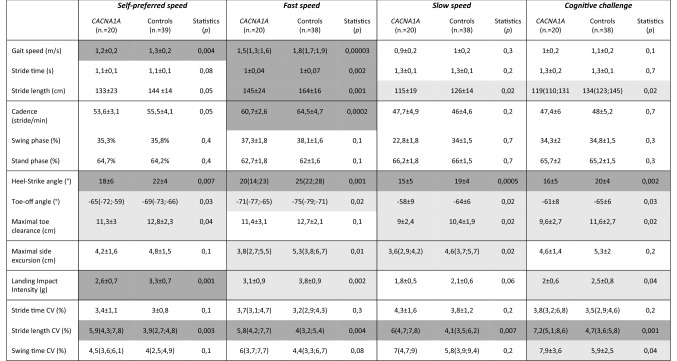
Descriptive data and statistics for between group comparisons for all gait variables in the four gait tasks

Data are reported either as mean ± SD or median(interquartile range)

Statistically significant comparisons are highlighted in grey (light grey < 0.05; dark grey < 0.01)

Patients displayed slower average gait speed compared to controls at self-determined preferred and fast speed, while at slow speed and cognitive dual task no significant differences were detected. Mean stride length was generally lower in the patients compared to controls. While the difference in mean stride length was only marginal at self-preferred speed, it became evident in the other tasks, especially at fast speed. Furthermore, an increased variability of this parameter (stride length CV in Table 3) distinguished the patients from the controls across all the tasks. The step width (reflected by the “maximal side excursion”) did not differ between patients and controls at self-determined preferred speed. The patients even displayed a slightly narrower base in the fast and slow speed tasks. The duration of the swing and stand phases was similar in patients and controls. A reduced ankle range of motion during lifting-up and strike was consistently observed in the patients group. Patients also displayed generally a lower landing acceleration.

Higher SARA scores correlated with reduced gait velocity (r(18) = − 0.49, *p* = 0.03), reduced toe-off angle (r(18) = 0.53, *p* = 0.02) and particularly reduced landing acceleration (r(18) =  − 0.57, *p* = 0.009) at self-determined preferred speed. Gait parameters did not differ between patients with and without nystagmus (data not shown). No significant differences were found among the three genotypes (EA2, FHM1, SCA6). Nonetheless, considering the episodic CACNA1A group (EA2 and FHM1) as opposed to SCA6, the first ones had a significantly narrower gait basis (maximal side excursion at self-preferred speed: 3.6 cm [2.7; 4] in EA2/FHM1 versus 4.9 cm [3.5; 5.8] in SCA6, *p* = 0.03). Moreover, the episodic CACNA1A group displayed a narrower gait basis also in the comparison with controls (*p* = 0.03 at self-preferred speed).

### Spatiotemporal gait parameters after challenge

**Fig. 2:**
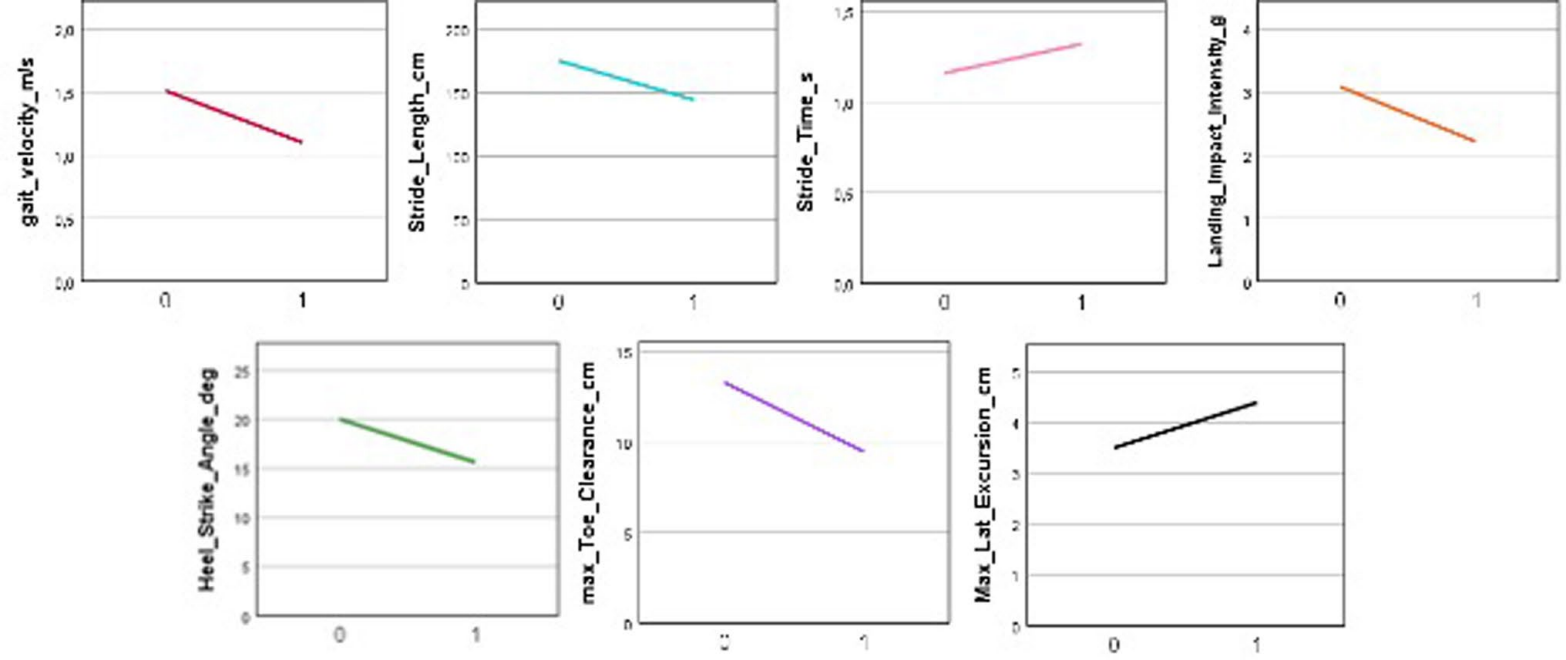
Changes in gait parameters before (= 0) and after (= 1) treadmill challenge in a EA2 patient with effort-triggered attack

Four patients with EA2 underwent a second IGA testing after 10 min cycling or walking on treadmill. In one of them, the physical effort triggered a mild episodic symptomatic with a worsening of the gait that was reflected by changes in the IGA parameters (see Fig. 2). In the other three patients, no substantial differences were observed both clinically and at IGA.

### Spatiotemporal gait parameters and falls

Five patients reported falls and two of them experienced fall-related injuries. Patients with history of falls displayed a tendency towards higher SARA scores (7.7 ± 4 versus 5.6 ± 4.8 in patients without falls, *p* = 0.3). Concerning the IGA parameters, no significant differences were found between patients with and without occurrence of falls.

## Discussion

In the present work, we applied a standardized IGA protocol using wearable sensors in a genetically confirmed *CACNA1A* cohort as a complementary testing to the routine clinical evaluation.

Consistent with previous studies in ataxic disorders [11], *CACNA1A* patients displayed lower average gait speed, shorter steps and increased stride length variability at IGA. *Contrasting* with the typical gait pattern of ataxic disorders, *CACNA1A* patients did not display a longer stand phase nor a widened gait base. Interestingly, the IGA detected an even narrower gait-width in *CACNA1A* patients as compared to controls. Further on, IGA consistently revealed a reduced range of ankle motion in *CACNA1A* patients across all gait tasks as well as a lower landing impact. In one patient experiencing mild episodic symptoms after physical exertion, the IGA was able to detect the deterioration over all gait parameters.

Globally, the present IGA findings reflect the mild stage of ataxia, but also unravel pathologic changes which would otherwise escape the clinical evaluation. For instance, a great impairment in ankle range of motion in Parkinson´s disease may reflect one aspect of the complex phenomenon of the “shuffling” gait [10]. In *CACNA1A* disorders, more subtle alterations, not related to a clinically evident shuffling but captured by IGA, may contribute to a reduced stability of locomotion and increased susceptibility to falls upon perturbations and may be the target of therapeutic interventions.

The detection of an overall non-widened-based gait in this cohort deserves further comments. Indeed, we detected an even narrower gait basis in the episodic CACNA1A disorders, as compared to controls and as opposed to the classical broadened-based gait in the SCA6 group. This finding is in accordance with our clinical impression. A wide base represents a natural compensatory mechanism which is highly sensitive for an underlying neurological disturbance but not specific, being detected in a variety of conditions affecting gait [12]. On the contrary, a narrow-based gait is uncommon but it can be seen in PD and in early stages of progressive supranuclear palsy [12]. The normal walking dynamics requires a constant shift of weight from the swing foot to the stance foot and to this concern a narrow-based walking requires smaller postural adjustments. For this reason, a narrow-based walking may be particularly advantageous in early-stage PD patients, who do not have an impairment of the mediolateral balance [13]. In progressive supranuclear palsy, a narrow-based gait is believed to result from the lack of perception of instability and reckless motor behavior, due to frontal dysfunction. Concerning our patients with EA2/FHM1, the high rate of falls compared to the ataxia stage, argues against a favorable impact of a narrow base on walking stability and rather points to a failure of the compensatory mechanisms, which would naturally lead to a widened-based gait. Imaging studies show that, differently from other ataxias, *CACNA1A* disorders display a mostly moderate cerebellar atrophy, which is usually more prominent—or event limited—to the vermis [6]. The cerebellar vermis displays tight connections with the pedunculopontine nucleus, a crucial supraspinal control center, and also with medial and lateral premotor areas [14, 15] The cortical projection to the vermis are believed to be part of the neural network involved in anticipatory postural adjustments and locomotion [15]. An abnormal functional connectivity of the vermis with the sensorimotor cortex has been recently shown to correlate with the impairment shown by the IGA parameters in PD [16]. In this view, the pattern of gait in *CACNA1A* disorders might reflect in the first place a defective connectivity in the higher control of gait through corticocerebellar projections [15].

The detection of a lower landing impact in *CACNA1A* patients represents a further interesting finding. Most research on this parameter derives from biomechanics analysis in runners, but it is also known that older people for example display a lower landing acceleration, *e.g.,* while descending stairs. This is believed to be a compensation mechanism for the poorer balance control, which is achieved by increased “stiffening” at knee and ankle of the stance leg [17]. We believe that this phenomenon may underlie the particular walking pattern, which in few of our patients results in a clinically evident cautious-seeming, heel-based gait, as if they were “walking on eggshells”.

These results demonstrate that abnormal.

vermal functional connectivity with sensorimotor cortex,

The preliminary observations from the post exertion testing suggest that wearable sensors may be applied in a home setting to monitor fluctuations, which are typical of paroxysmal *CACNA1A* disorders, but are often subjected to significant recall bias.

To the best of our knowledge, no biomarker or outcome measure has been systematically assessed in respect to an application in clinical trials in EA2 and FHM1. Concerning SCA6, a European natural history study showed that the SARA score increases linearly with the disease progression, though extremely slowly (0.8 points/year) [18]. Using the SARA as outcome measure, 302 patients would be needed for a 2-year clinical trial in SCA6. Of note, the SARA evaluates not only gait ataxia and the extremities items (finger-chase, heel-shin test) have a major impact on the final score. Since the gait disorder is the major ataxia manifestation and a major source of disability in *CACNA1A* patients, the definition of gait-specific, more sensitive biomarkers/outcome measures is urgently needed in the view of future clinical trials.

In all, evidence from systematic studies on gait disturbances in *CACNA1A* disorders is poor. Rochester and colleagues evaluated by means of an instrumented mat 24 SCA6, of whom six presymptomatic [19]. Patients with overt disease differed from the controls in all parameters, while the presymptomatic displayed solely increased variability in the step-width and -time. Furthermore, a case series on two patients with *CACNA1A* mutations showed an improvement of stride time variability, evaluated with a gait mat system, upon treatment with 4-aminopyridine [20]. Our study extends the current knowledge offering a first systematic characterization of gait in a larger group of paroxysmal *CACNA1A* disorders. Importantly, we demonstrated that IGA is able to detect subtle gait changes which are not manifest to the clinical naked eye and correlated with the clinical rating scores. Comparing to other technologies, wearable systems can measure some parameters only indirectly (for example the medio-lateral excursion) and often require complex algorithms to extract the data [11]. Nonetheless, wearable sensors are easy to use, suitable in the clinical practice and can be potentially applied also for remote motion analysis in a home monitoring setting. These findings support the usefulness of wearable sensors as ancillary devices in the characterization, diagnostic, follow-up or outcome quantification of gait impairments in *CACNA1A* spectrum disorders. A longitudinal evaluation as well as a home-monitoring phase are currently planned.

In conclusion, the application of a standardized IGA protocol allowed us to define specific gait signatures of *CACNA1A* disease spectrum and has relevance for the design of clinical trials and physical therapy interventions.

## Data Availability

The dataset of the present study is available upon request.
